# Gut microbiomes of bigheaded carps and hybrids provide insights into invasion: A hologenome perspective

**DOI:** 10.1111/eva.13152

**Published:** 2020-12-22

**Authors:** Lifeng Zhu, Zheng Zhang, Hua Chen, James T. Lamer, Jun Wang, Wenzhi Wei, Lixia Fu, Minghu Tang, Chenghui Wang, Guoqing Lu

**Affiliations:** ^1^ College of Life Sciences Nanjing Normal University Nanjing China; ^2^ Mingke Biotechnology Center Hangzhou China; ^3^ Department of Biological Sciences Western Illinois University Macomb IL USA; ^4^ Key Laboratory of Freshwater Fisheries Germplasm Resources Ministry of Agriculture and Rural Affair/National Demonstration Center for Experimental Fisheries Science Education/Shanghai Engineering Research Center of Aquaculture Shanghai Ocean University Shanghai China; ^5^ College of Animal Science and Technology Yangzhou University Yangzhou China; ^6^ Yangzhou Hanjiang National Carp Seed Farm Yangzhou China; ^7^ Department of Biology University of Nebraska at Omaha Omaha NE USA

**Keywords:** alpha and beta diversity, bigheaded carps, food resource utilization, gut microbiome, hybrids, invasion

## Abstract

Gut microbiomes play an essential role in host survival and local adaptation and thus can facilitate the invasion of host species. Biological invasions have been shown to be linked to the genetic properties of alien host species. It is thus plausible that the holobiont, the host, and its associated microbiome act as an entity to drive invasion success. The bighead carp and silver carp (bigheaded carps), invasive species that exhibit extensive hybridization in the Mississippi River Basin (MRB), provided a unique model to test the holobiont hypothesis of invasion. Here, we investigated the microbiomes of foreguts and hindguts in bigheaded carps and their reciprocal hybrids reared in aquaculture ponds using 16S amplicons and the associated gene prediction. We found an admixed pattern in the gut microbiome community in bigheaded carp hybrids. The hybrid gut microbiomes showed special characteristics such as relatively high alpha diversity in the foregut, an increasing dissimilarity between foreguts and hindguts, and a remarkable proportion of genes coding for putative enzymes related to their digestion of main food resources (*Cyanobacteria*, cellulose, and chitin). The pond‐reared hybrids had advantageous features in genes coding for putative enzymes related to their diet. The above results collectively suggested that the gut microbiomes of hybrids could be beneficial to their local adaptation (e.g., food resource utilization), which might have facilitated their invasion in the MRB. The gut microbial findings, along with the intrinsic genomic features likely associated with life‐history traits revealed in our recent study, provide preliminary evidence supporting the holobiont hypothesis of invasion.

## 1 INTRODUCTION

The hologenome concept asserts that the holobiont, or the host with all of its associated microbiomes, functions as the primary entity of selection in evolution (Rosenberg & Zilber‐Rosenberg, 2018). It has been found that the distinct gut microbiomes of closely related *Nasonia* wasp species contribute to the death of hybrids, providing evidence of interactions between a host and its symbiont microbiome (Brucker & Bordenstein, 2013). Many studies have indicated that microbiomes play essential roles in host fitness (Ley et al., 2008; Zilber‐Rosenberg & Rosenberg, 2008) and local adaptation to new environments (Dulski et al., 2020; Lefort et al., 2017; Rennison et al., 2019; Wang et al., 2019). The observation of common traits in the gut microbiomes of invasive insects suggests that the gut microbiome could be a critical determinant of invasion success (Lefort et al., 2017). However, this hypothesis has not been widely tested in species other than herbivorous insects. The characteristics, both biotic and abiotic, of alien host species and the environments where they have been introduced also strongly influence the establishment and colonization of these species and whether they become invasive.

There are many putative mechanisms that enable the success of invasive species, including high genetic diversity (Dlugosch & Parker, 2008; Roman & Darling, 2007), an increased reproductive rate (Clark et al., 2001; Huxel, 1999; Traveset & Richardson, 2006), active foraging (Bøhn et al., 2008; Ficetola et al., 2012), and hybridization (Ellstrand & Schierenbeck, 2000; Figueroa et al., 2003; Huxel, 1999; Mooney & Cleland, 2001; Wang et al., 2020). Bighead carps (*Hypophthalmichthys nobilis*) and silver carps (*Hypophthalmichthys molitr*; bigheaded carps) belong to the family *Cyprinidae* and are native to East Asia (Li et al., 1989). Both species were introduced to the United States in the 1970s and have become successful invasive species in North America (Kolar et al., 2005). Our recent study has identified intrinsic genomic features in these fish such as high heterozygosity and more genes related to environmental adaptation and feeding habits compared to other fish species. These features suggest that the genomes of bigheaded carps might have facilitated their early establishment in the Mississippi River Basin (Wang et al., 2020). Extensive introgressive hybridization between invasive bighead carp and silver carp has been reported in the Mississippi River Basin (MRB; Lamer et al., 2015, 2019). The findings of high genomic similarity between bighead and silver carps and over 90% embryonic viability in all crosses suggest interspecific hybridization between the carps might have promoted their range expansion (Wang et al., 2020). These genomic findings do not rule out the possible significance of gut microbiomes in facilitating the invasion of bigheaded carps.

Bighead and silver carps are large‐bodied planktivorous species (Ke et al., 2008; Tumolo & Flinn, 2019). Bighead carp consume more zooplankton than silver carp (Cooke et al., 2009; Ke et al., 2008). A high proportion of *Cyanobacteria* was found in the gut microbiome of invasive silver carp in the Mississippi River Basin, indicating that their major food source is green algae (Ye et al., 2014). A gut microbiome study by Eichmiller et al. (2016) found that the gut microbial communities of silver and bighead carps were dissimilar to some extent, which may be caused by phylogenic and dietary factors. The local environments were suggested to be a dominant factor shaping the gut bacterial communities of invasive carps (Eichmiller et al., 2016). Host genomic interactions have been suggested to have an effect on shaping the gut microbiome in reciprocal hybrid fish (Li et al., 2018; Sevellec et al., 2018, 2019). We sought to explore the role of gut microbiomes of bigheaded carps in their successful invasion.

Species hybridization involves the genomic admixture of parental species in hybrids, which has been employed to investigate interactions between host genomes and the gut microbiome (Li et al., 2018; Sevellec et al., 2018, 2019). The gut microbiome analysis of two house mice subspecies and their hybrids showed widespread transgressive phenotypes in a variety of aspects of bacterial community structure (Wang et al., 2015). The hybrids displayed genetic incompatibilities, aberrant immune gene expression, and increased intestinal pathology associated with altered community structure, which confirms the consequences of evolutionary divergence in a vertebrate hologenome (Wang et al., 2015). In lake whitefish, including hybrids reared in a laboratory setting, the complex interactions between the host, the microbiome, and the environment suggested three distinct evolutionary paths in the intestinal microbiome (Sevellec et al., 2019). The gut microbiomes of hybrids between herbivorous blunt snout bream (*Megalobrama amblycephala*) and carnivorous topmouth culter (*Culter alburnus*) suggested that host genomic interaction (mainly subgenome domination) had a sizeable effect on shaping gut microbiomes in reciprocal hybrid fish (Li et al., 2018).

This study was designed to test the hologenome hypothesis of invasion; that is, the host genome and its gut microbiome act as a primary entity in driving invasion success. Bigheaded carps (silver carp and bighead carp) and their hybrids provided a robust system to investigate the possible association of gut microbiomes with successful invasions. Given that different gut sections have distinct functionality (Stevens & Hume, 1998), we investigated gut microbiomes in both foreguts and hindguts. We sought to determine whether the gut microbiome in hybrids harbored a mosaic pattern in community composition and structure and had relatively higher alpha diversity compared to the parent species. The gut microbial features likely associated with feeding habits, an important attribute related to invasion, were also examined. This study is expected to provide insights into the invasion mechanisms based on gut microbiomes, and the findings could assist in the development of better strategies in the management and control of invasive bigheaded carps.

## 2 MATERIALS AND METHODS

### 2.1 Ethical statement

The experiments were approved by the Animal Care and the Ethics Committee of Shanghai Ocean University.

### 2.2 Sample collection

We conducted a hybridization experiment with bigheaded carps in 2012 as described in detail in Wang et al. (2020). Fingerlings of bigheaded carps and their hybrids were tagged with integrated transponders (PIT) and cultured in three aquaculture ponds. Fish samples (~2 years old) were randomly collected for gut bacterial analysis and growth performance comparison. Growth‐related characters included body weight (g), body length (mm), body height (mm), and body width (mm). A one‐way ANOVA test was conducted in SPSS Statistics 20.0 (Spss, 2011) to detect the significance of differences in growth performance‐related parameters. Multiple comparisons among groups were conducted by an LSD (least significant difference) test at a significant level 0.05 in SPSS Statistics 20.0 (Spss, 2011).

For each fish, we collected gut contents from two gut sections, that is, the foregut (F) and hindgut (H) of bighead carp (B), silver carp (S), hybrids of bighead (female) and silver (male) carp (BS), and hybrids of silver (female) and bighead (male) carp (SB) reared in aquaculture ponds in 2014 (Table S1). Each fish was dissected using sterile scalpels and scissors. The gut content was squeezed into a 15‐ml sterilized tube and shipped to the laboratory using dry ice. The samples were preserved in a −80°C freezer.

### 2.3 DNA extraction and sequencing

We used a QIAamp DNA Stool Mini Kit (Qiagen) to extract DNA from the gut contents and also used a blank control (negative control including kit, but not any gut contents) to avoid extraction contamination. The V4 region of the 16S ribosomal RNA gene was amplified with 515F (5′‐GTGCCAGCMGCCGCGGTAA‐3′) and 806R (5′‐GACTACHVGGGTWTCTAAT‐3′) primers (Caporaso et al., 2012). The PCR was conducted using the following conditions: 95°C for 5 min, 35 cycles of 95°C for 30 s, 55°C for 30 s, and 72°C for 45 s, with a final extension step at 72°C for 10 min. All of the PCR products from the blank controls were blank in the agarose gel. We did not sequence the negative control. Sequencing libraries were prepared according to the MiSeqTM Reagent Kit Preparation Guide (Illumina). Sequencing was conducted using the Illumina MiSeq platform.

### 2.4 16S rRNA gene‐based sequence analysis

The quality control of sequence reads was performed according to the following steps: (a) the *search* function was used to remove the chimeric sequences and low‐quality sequences; (b) the *flash* function was used for splicing; and (c) the *trimmomatic* function was used for quality control based on default parameters (e.g., window size: 20 base pair; minimum read length: 50 base pair; Edgar, 2010). The ratio of the total number of high‐quality sequences to the total number of raw sequences was about 0.8. The high‐quality sequences were used to identify operational taxonomic units (OTUs) by searching the SILVA132 database with a cutoff value of 97% sequence identity (Quast et al., 2013) in QIIME 1.9 (Caporaso et al., 2010). We used QIIME 1.9 (Caporaso et al., 2010) for the classification analysis of taxonomic groups based on the OTU table. We rarefied sequencing depth at 5,000 sequences per sample (according to the lowest number of sequences among samples in this study) to decrease the bias caused by sequencing. Given that we did not sequence the negative control, we used decontam (https://benjjneb.github.io/decontam/) to detect well‐known contaminants, and only found one rare OTU (only having two sequences in all samples) out of 1,732 OTUs that may have been a putative contaminant. Thus, this finding further confirmed the reliability of our 16S dataset with the negative control in experiment and quality control in the raw dataset.

### 2.5 Gut microbiome composition between bigheaded carps and hybrids

We estimated the mean abundance for each gut bacterial phylum and genus in the foreguts and hindguts of for each species group. First, we used LEfSe (linear discriminant analysis Effect Size; Segata et al., 2011) to determine the gut microbial taxa with significantly differentiating abundance within the same gut section among groups. The default LDA score was 3, and the significant level was at 0.05. Second, we used Welch's *t* test (*p*‐value corrected) in STAMP (Parks et al., 2014) to determine the significance of differences in the mean phylum abundances between foreguts and hindguts within each group.

### 2.6 Alpha diversity of gut bacterial communities and differences among groups

The alpha diversity (the phylogenetic diversity [PD]) was estimated using QIIME 1.9 (Caporaso et al., 2010). We used a one‐way ANOVA test in SPSS Statistics 20.0 (Spss, 2011) to test whether the differences in alpha diversity between groups were significant. We then selected the Dunn–Sidàk correction to make a strict and conservative *p*‐value correction to test the significant difference among groups (Abdi, 2007). The *t* test in SPSS Statistics 20.0 (Spss, 2011) was used to compare the difference in mean values of alpha diversity of bacterial communities between foreguts and hindguts within the group.

### 2.7 Beta diversity analysis of gut bacterial communities among bigheaded carps

We used the Adonis nonparametric statistical method in QIIME 1.9 (Caporaso et al., 2010) to compute an *R* ^2^ value (effect size) and evaluate the significance of defined categories based on unweighted UniFrac distances (Lozupone & Knight, 2005) using the OTU table. The defined categories were species groups (B, S, BS, and SB), gut sections (foreguts and hindguts), and gut sections within the group. We used the PCoA function and Adonis test in QIIME 1.9 (Caporaso et al., 2010) to evaluate the effect of these defined categories based on unweighted UniFrac distances. The categories included groups (different species) and gut sections (foregut and hindgut). The one‐way ANOVA was used to test significant differences among the pairwise unweighted UniFrac distance between the foregut and hindgut within each group. If significant, we selected the Dunn–Sidàk correction to make multiple comparisons (Abdi, 2007).

### 2.8 Functional prediction of gut bacterial communities

We used tax4fun (Aßhauer et al., 2015) to predict the functions of gut bacterial communities. Tax4fun predicts metagenomes using 16S‐based OTU tables. Predictions were conducted based on annotated genomes across all KEGG (Kyoto Encyclopedia of Genes and Genomes) orthologs (KOs). The resulted KOs mapped to KEGG pathways were used to estimate the relative abundance of enzymes for each sample (Aßhauer et al., 2015). Based on the literature concerning the main food sources of bigheaded carps (Cooke et al., 2009; Ke et al., 2008; Tumolo & Flinn, 2019), we focused on enzymes involved in the digestion of *Cyanobacteria* (cyanophycinase, degrading cyanophycin, a water‐insoluble reserve material of *Cyanobacteria*; Richter et al., 1999), cellulose (cellulose 1,4‐beta‐cellobiosidase, endoglucanase, and beta‐glucosidase), and chitin (chitinase and chitin‐binding protein). The one‐way ANOVA test was conducted in SPSS Statistics 20.0 (Spss, 2011) to detect the significance of differences in the mean proportion of coding genes for the putative enzymes involved in the digestion of main food sources among different groups of bigheaded carps. If significant, we selected the Dunn–Sidàk correction to make multiple comparisons (Abdi, 2007).

## 3 RESULTS

### 3.1 The differences in the gut microbiome composition between bigheaded carps and hybrids

**FIGURE 1:**
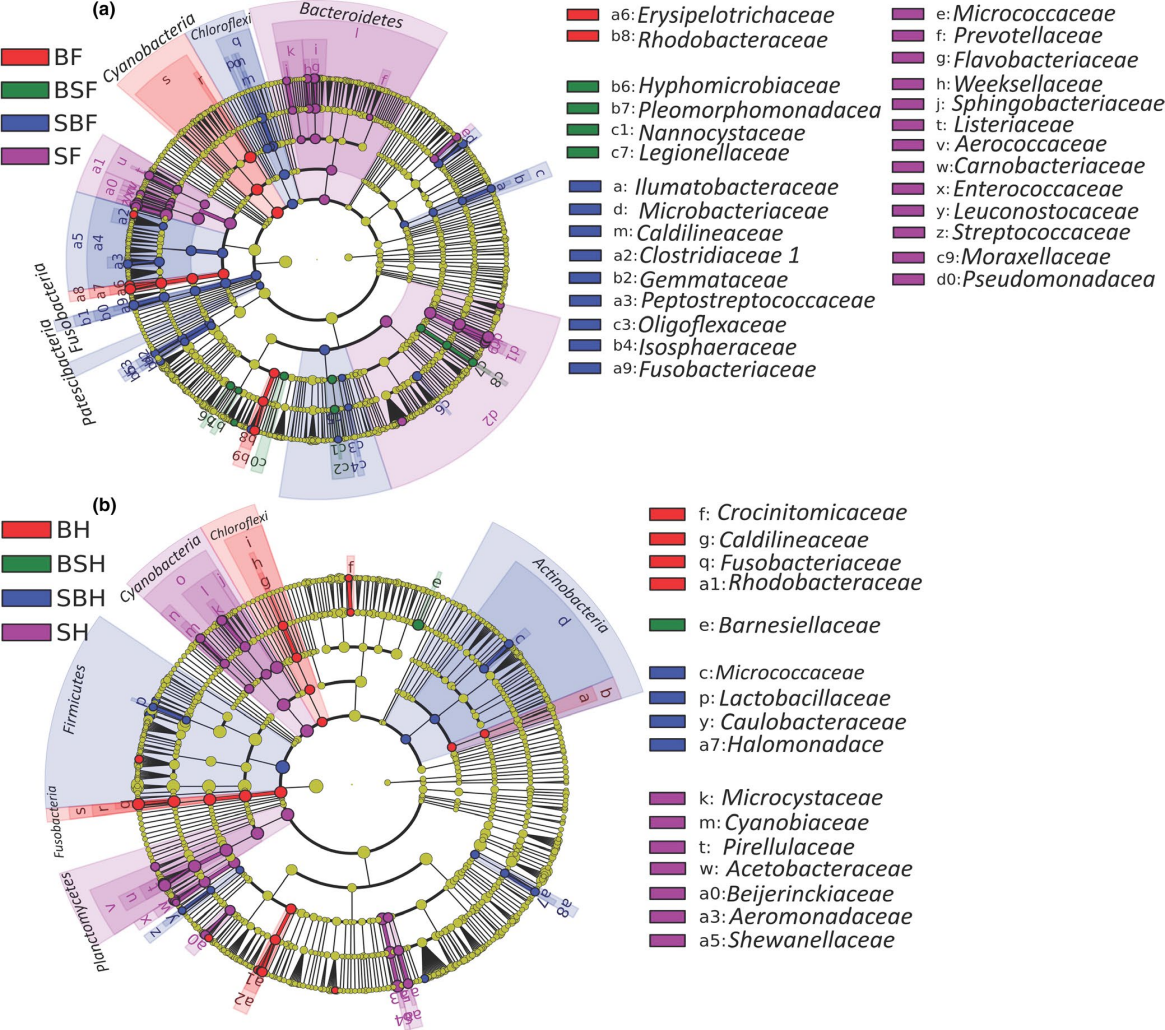
The LEfSe (linear discriminant analysis effect Size) analysis in the gut microbiome community (family level) within the same gut section among groups. (a) The foregut. (b) The hindgut section. B, S, BS, and SB represent bighead carp, silver carp, hybrids of bighead (female) and silver carps (male), and hybrids of silver (female) and bighead Carps (male), respectively, whereas F and H denote foreguts and hindguts, respectively

We obtained 16S rRNA sequencing data from 170 samples. These samples comprised 49, 37, 39, and 45 from pond‐reared bighead carp (B), silver carp (S), bighead and silver hybrids (BS), and silver and bighead hybrids (SB), respectively (Table S1). The domain phyla in bigheaded carps included *Firmicutes*, *Proteobacteria*, *Cyanobacteria*, *Planctomycetes*, *Fusobacteria*, and *Bacteroidetes* (Figure S1). LEfSe (Segata et al., 2011) analysis showed that the gut microbial taxa with significantly differentiating abundance within the foregut among the groups mostly occurred in hybrid SB and silver carps (S; Figure 1a). The microbial taxa from *Fusobacteriaceae* (belonging to *Fusobacteria)* and *Clostridiaceae* (belonging to *Firmicutes*) were significantly enriched in hybrid SB foreguts, and the microbial taxa from *Bacteroidetes* were significantly enriched in silver carp foreguts. The gut microbial taxa with significantly differentiating abundance within the hindgut among groups mostly occurred in hybrid SB, bighead carps (B), and silver carps (S; Figure 1b), while only one microbial taxon (*Barnesiellaceae*) was significantly enriched in hybrid BS (Figure 1b). These findings indicated a difference in the gut microbiome composition between the bigheaded carps and hybrids and even between the hybrid BS and SB.

We used one‐way ANOVA to test for significant differences in the dominant microbial phyla within the same gut section among groups and found that the abundances of *Bacteroidetes*, *Planctomycetes*, and *Cyanobacteria* in foreguts were significantly different among groups (Figure S2). The abundances of *Firmicutes*, *Planctomycetes*, and *Cyanobacteria* were significantly different in hindguts among groups. Compared to B and S, the hybrid groups (BS and SB) had intermediate abundances of *Cyanobacteria* and *Bacteroidetes* in the foregut (Figure S2). The abundance of *Cyanobacteria* in silver carp foreguts was significantly higher than that in bighead carps (Table S3). The abundance of *Bacteroidetes* in the silver carp foreguts was significantly higher than those in the other three groups (Table S3).

**FIGURE 2:**
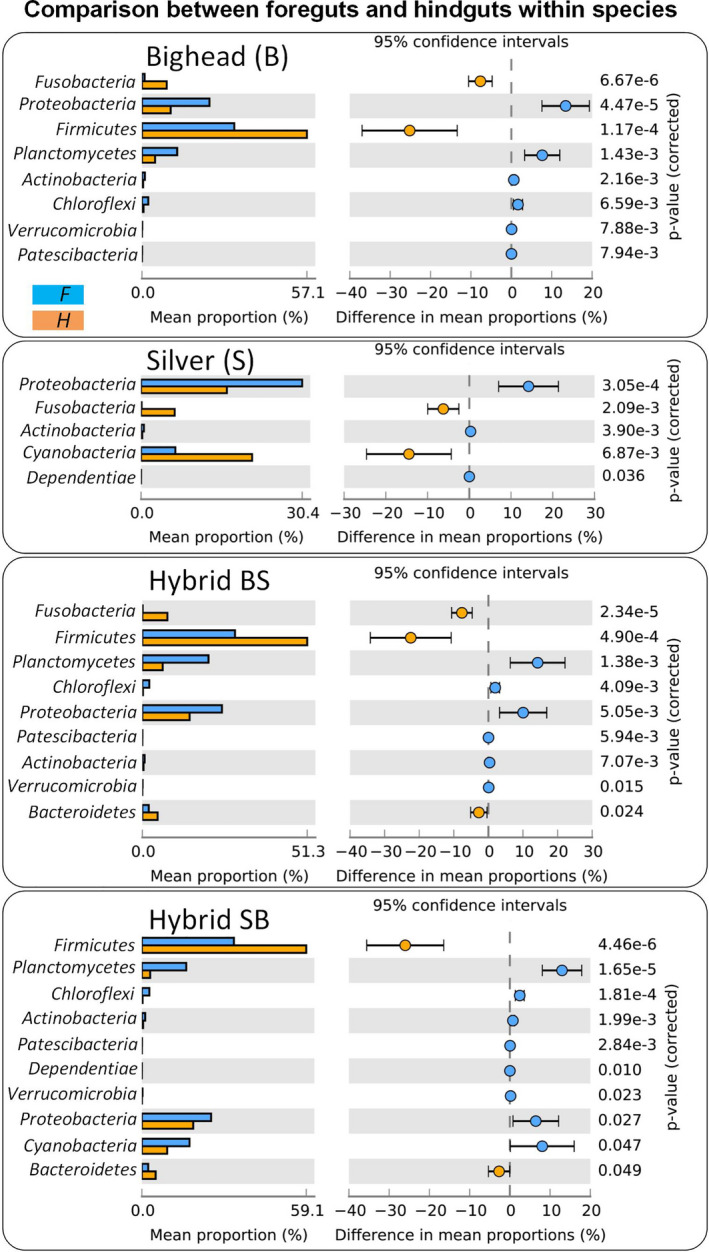
Abundances of major bacterial phyla and significant differences between foreguts and hindguts in bigheaded carps. Welch's *t* test (*p*‐value corrected) was used to determine the significance of differences in the mean phylum abundances between foreguts (F) and hindguts (H) within each group. B, S, BS, and SB represent bighead carp, silver carp, hybrids of bighead (female) and silver carps (male), and hybrids of silver (female) and bighead carps (male), respectively

We then compared the significant differences in the microbiome composition between the foregut and hindgut within the same group (Figure 2). The foregut of the carps harbored relatively high abundances of *Cyanobacteria*, especially in SB hybrids. The *Planctomycetes* in foreguts were found to be significantly enriched in B, BS, and SB. *Proteobacteria* in foreguts were found to be enriched in most bigheaded carps. In the hindgut, *Fusobacteria* were significantly enriched in the bigheaded carps (B and S), and *Firmicutes* were significantly enriched in B, BS, and SB. These findings indicated the potential patterns of differences in the microbiome composition between the different gut sections between the bigheaded carps and hybrids.

### 3.2 The differences in the phylogenetic diversity of gut microbiome between bigheaded carps and hybrids

**FIGURE 3:**
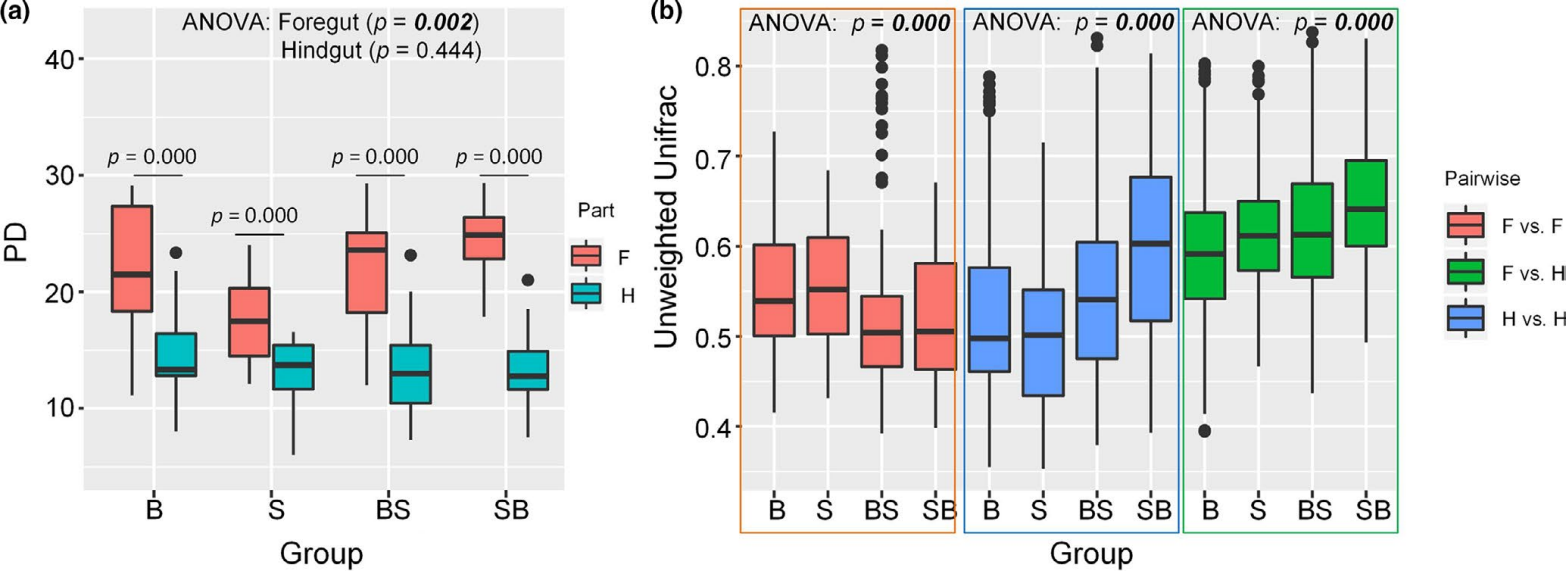
The boxplots of alpha and beta diversity of gut bacterial communities in each species group. (a) The phylogenetic diversity (PD). The *p*‐value referred to the Pairwise *t* tests between foreguts (F) and hindguts (H) within each group. (b) Pairwise unweighted UniFrac distances between bacterial communities of F and H within each group. B, S, BS, and SB represent bighead carp, silver carp, hybrids of bighead (female) and silver carps (male), and hybrids of silver (female) and bighead carps (male), respectively, whereas F and H denote foreguts and hindguts, respectively. A one‐way ANOVA test was used to compare the difference in the mean of pairwise distance among groups

The PD indices of bacterial communities were significantly higher in foreguts than those in hindguts in most groups (Figure 3a). The PD indices of bacterial communities were significantly different among the foreguts of bigheaded carps, and the PD indices in foreguts of the hybrid fish were relatively higher than those in pond‐reared bighead and silver carps (Figure 3a). We found that the PD indices in the foreguts of hybrid SB were significantly higher than those in silver carps (Dunn–Sidàk correction, *p* = .001).

### 3.3 High variation in gut microbial communities of hybrids between the foregut and hindgut

The pairwise comparison of foregut bacterial communities (*F v F*) within‐group showed that the unweighted UniFrac distance was lower within hybrids (BS or SB) than that within bigheaded carps (B or S; Figure 3b). The pairwise comparison of hindgut bacterial communities (*H v H*) within‐group showed that the unweighted UniFrac distance was higher within hybrids (BS or SB) than that within bigheaded carps (B or S; Figure 3b). The pairwise comparisons within hindguts and between foreguts and hindguts (*F v H*) within‐group showed that the unweighted UniFrac distances of bacterial communities in hybrid groups (especially in SB) were significantly higher than that in B or S, indicating a higher variation in gut bacterial communities between the gut sections in hybrid carps compared to those in bigheaded carps (Table 1).

**TABLE 1:** Multiple comparisons (Dunn–Sidàk correction) among the pairwise unweighted UniFrac distance between the foregut and hindgut within each group

	(I) Group	(J) Group	The difference in mean (I–J)	*SEM*	*p*‐value
Dunn–Sidàk correction	B	S	−0.02405*	.00513	.000
		BS	−0.03460*	.00473	.000
		SB	−0.06013*	.00435	.000
	S	B	0.02405*	.00513	.000
		BS	−0.01055	.00558	.305
		SB	−0.03608*	.00526	.000
	BS	B	0.03460*	.00473	.000
		S	0.01055	.00558	.305
		SB	−0.02553*	.00487	.000
	SB	B	0.06013*	.00435	.000
		S	0.03608*	.00526	.000
		BS	0.02553*	.00487	.000

Note

The one‐way ANOVA test showed a significant difference among the pairwise unweighted UniFrac distance between the foregut and hindgut within each group. Then, we selected the Dunn–Sidàk correction to make multiple comparisons. B, S, BS, and SB, represent bighead carp, silver carp, hybrids of bighead (female) and silver carps (male), and hybrids of silver (female) and bighead carps (male), respectively. *, significant at level 0.001.

**FIGURE 4:**
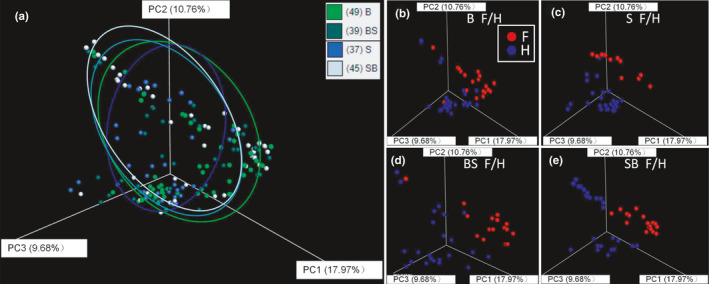
PCoA clustering of gut bacterial communities using unweighted UniFrac distance among different groups. (a–e) show the PCoA clustering of gut microbiomes in different species groups (a), pond‐reared bighead carp (b), pond‐reared silver carp (c), pond‐reared hybrids of bighead and silver Carps (d), and pond‐reared hybrids of silver and bighead carps (e), respectively, whereas F and H denote foreguts and hindguts, respectively

The Adonis variance analysis using unweighted UniFrac distance based on total samples showed that species groups (B, S, BS, and SB) and gut sections (foregut, F and hindgut, H) had significant effects on gut bacterial communities (Figure 4a; Table 2). The PCoA clustering of gut bacterial communities using unweighted UniFrac distances displayed an admixed pattern between pond‐reared bigheaded carps and hybrids (Figure 4a). Within each group, the gut sections (F versus H) had a significant effect on bacterial communities (Figure 4b–e), which was consistent with the above finding, that is, pairwise distances between foreguts and hindguts were the highest compared to those within foreguts or hindguts within each species or hybrid group (Figure 3b).

**TABLE 2:** The Adonis results in evaluating the effect of these defined categories based on unweighted UniFrac distances

Categories	*df*	*F*	*R* ^2^	*p*
Species	3	1.990	.188	.001
B_Gut part	1	5.264	.101	.001
S_Gut part	1	6.427	.155	.001
BS_Gut part	1	7.077	.160	.001
SB_Gut part	1	7.827	.154	.001

Note

The categories included groups (different species), and gut sections (foregut and hindgut). B, S, BS, and SB, represent Bighead Carp, Silver Carp, hybrids of Bighead (female) and Silver Carps (male), and hybrids of Silver (female) and Bighead Carps (male), respectively.

### 3.4 Functional differences of gut bacterial communities between bigheaded carps and hybrids

**FIGURE 5:**
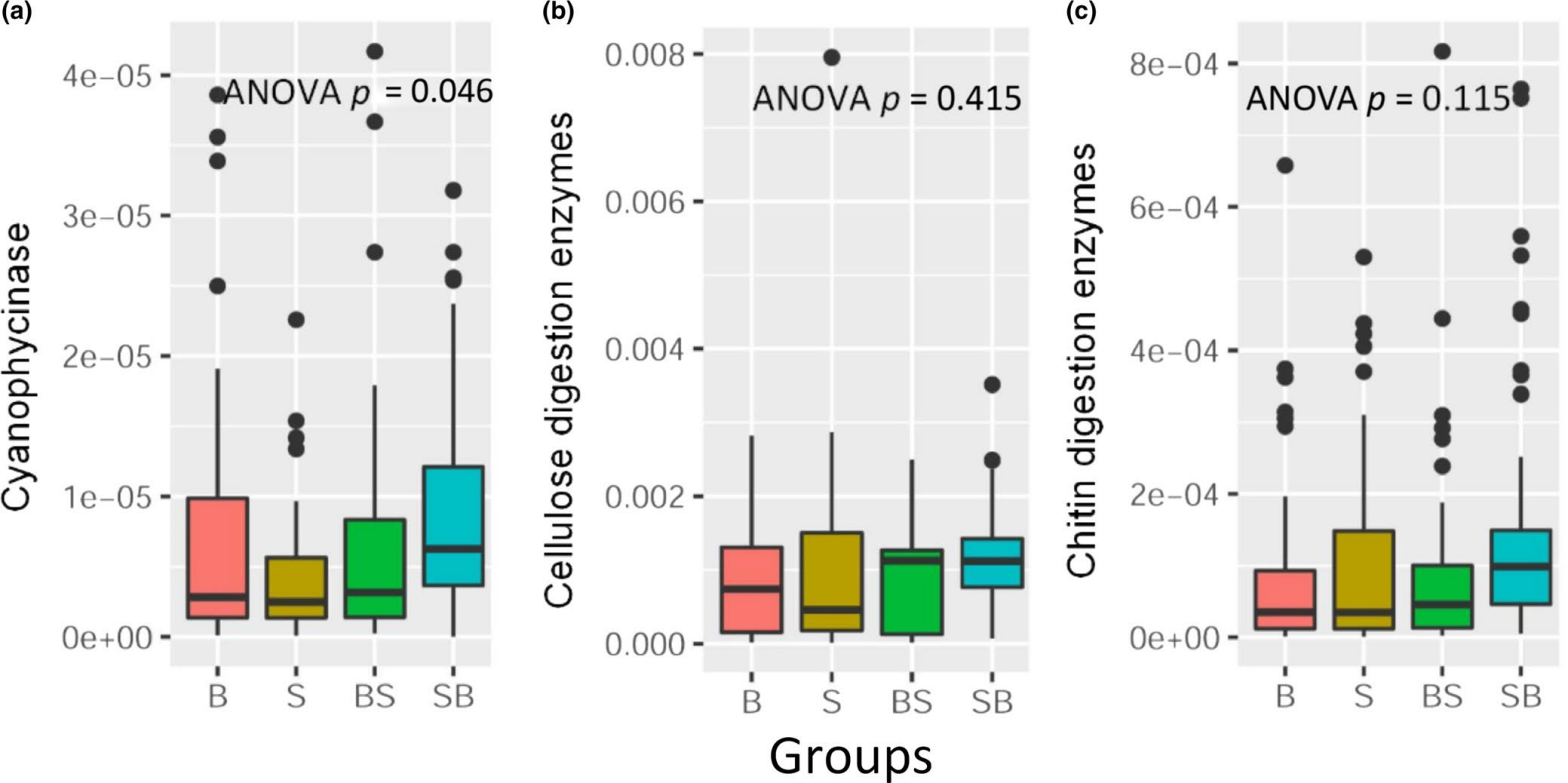
The boxplots of the proportions of coding genes for putative digestive enzymes in gut bacteria communities and the significance of differences among groups. (a) The proportion of genes coding for putative cyanophycinase involved in *Cyanobacteria* degradation for each species group. (b) The proportion of the genes coding for putative cyanophycinase involved in *Cyanobacteria* degradation in foreguts and hindguts within each species group. (c) The proportion of genes coding for putative enzymes involved in cellulose digestion for each species group. The one‐way ANOVA test was used to test the significant difference among groups. B, S, BS, and SB represent bighead carp, silver carp, hybrids of bighead (female) and silver carps (male), and hybrids of silver (female) and bighead carps (male), respectively, whereas F and H denote foreguts and hindguts, respectively

Bigheaded carps are planktonic filter‐feeders. We thus estimated mean abundances of putative enzymes related to the use of *Cyanobacteria*, cellulose, and chitin in the gut bacterial community. There was a significant difference in the mean proportion of genes coding for putative cyanophycinase among species (Figure 5a), and the mean abundances in hybrids (BS and SB) were higher than those in pond‐reared bigheaded carps. The abundance of genes coding for this enzyme in SB was significantly higher (Dunn–Sidàk correction, *p* = .030) than that in S. Overall, the mean abundances of genes coding for these three enzymes in SB were the highest among groups (Figure 5).

### 3.5 Comparison of growth performances among bigheaded carps and their hybrids

There was a significant difference in growth performances among bigheaded carps and their hybrids reared in aquaculture ponds, and the corresponding values were significantly lower in bighead carp compared to silver carp and hybrid carps (LSD, a significant level at 0.05; Figure S3a–c). The growth performances (e.g., body weight, length, and height) of hybrids were intermediate between bighead carp and silver carp. There was no significant difference in the body width among bigheaded carps and their hybrids (Figure S3d).

## 4 DISCUSSION

### 4.1 Microbiome difference among different species and gut sections

Investigating gut microbiomes is becoming increasingly important in understanding host fitness and adaption (De Schryver & Vadstein, 2014; Videvall et al., 2018; Zilber‐Rosenberg & Rosenberg, 2008). Numerous gut microbial studies across a diverse array of animals have yielded several general conclusions, and bigheaded carps are no exception. First, gut microbial community compositions are distinct among different species, even those cohabiting in the same environment (Baldo et al., 2017; Eichmiller et al., 2016; Ghanbari et al., 2015; Li et al., 2015). Second, species appear to possess core gut microbial communities, although variations exist that often reflect the living environments (Franchini et al., 2014; Sevellec et al., 2019). Third, different gut sections encompass dissimilar microbial communities with specific functions (Han et al., 2016; Looft et al., 2014; Videvall et al., 2018). Fourth, environments play a large role in shaping gut microbial community composition and structure (Eichmiller et al., 2016; Ghanbari et al., 2015; Sevellec et al., 2019). Thus, we speculated that the holobiont and its complex interaction with the environment might lead to the formation of distinct gut microbiomes in different species.

Bighead and silver carps (bigheaded carps) are the closest sister groups in the family *Cyprinidae* (Li et al., 1989). Here, based on the pond‐reared experiment, we found some transitional patterns in the gut microbiome community between the bigheaded carps and hybrids. For example, the mean abundance of *Cyanobacteria* and *Bacteroidetes* in the foregut of hybrids was within the range between bighead and silver carps. However, the hybrids also had some special features in gut microbiome composition. The mean abundance of *Planctomycetes* in the foregut of hybrids was higher than in the bighead and silver carps. *Planctomycetes* are widely distributed in the aquatic environment, even in wastewater habitats, and play an important role in the ammonium oxidation process (Jetten et al., 2001; Neef et al., 1998). Thus, the gut microbiome of hybrids had some common features compared to those in the bigheaded carps, but also had some distinct characteristics. These special features in hybrids might be associated with their fitness and local adaption (e.g., food digestion).

### 4.2 Gut microbiomes in hybrids and contribution to the invasion

Our previous cross experiment (Wang et al., 2020) and the observation of a high proportion of hybrids and their offspring (several generations) in the MRB (Lamer et al., 2015, 2019) indicated the hybrids of bigheaded carps can survive, grow well, and reproduce in the invaded region. In this study, we found an admixed pattern in the gut microbiomes of hybrid bigheaded carps (BS and SB), with certain special characteristics. The hybrids (especially SB) possessed a relatively high alpha diversity in foreguts, an increasing dissimilarity between foreguts and hindguts, and an elevated proportion of putative genes coding for putative enzymes related to the digestion of filter‐feeding phytoplankton (*Cyanobacteria*, cellulose, and chitin). Here, these were only predictions and not observed genes in the actual samples.

The growth performances of the hybrids were intermediate between silver and bighead carps reared in aquaculture ponds. In our cross experiment, we found that all crosses of bigheaded carps had a high fertilization rate and comparable high embryonic viability (Wang et al., 2020). The comparison of draft genomes revealed a high genomic similarity between bigheaded carps, with the majority of benign nonsynonymous SNPs in hybrids (Wang et al., 2020).

The genomic compatibility between the bighead and silver carps is one of the possible reasons for their hybridization and the normal health of the hybrids. The gut microbiome showed some special characters (high alpha diversity and potential advantages in food utilization) that would be beneficial for the survival, local adaptation, and invasion of hybrid bigheaded carps. It was noted that in the MRB, environmental factors such as less predation, low fishing efforts, and abundant food resources may also contribute to the success of invasive bigheaded carps. In this study, we combined the bigheaded carp genomes and their microbiota data in a coherent analysis to examine host genomic variants associated with gut microbiota profiles.

## 5 CONCLUSIONS

This study provides novel microbial insights into the fish hybrid as an ideal model for vertebrate hologenome research. We presented the gut microbiomes of hybrid bigheaded carps and compared gut microbiomes of bigheaded carps in different gut sections. We found distinct bacterial community structure and diversity between gut sections and among different groups of species (B, S) and hybrids (SB, SB). We explored the link between gut bacterial composition and feeding habits in bigheaded carps and their hybrids. The gut microbiomes, along with host genomes (Wang et al., 2020), may synergistically contribute to the role of bigheaded carps as the most important species in global aquaculture and the most notorious invasive species in the United States.

## CONFLICT OF INTEREST

None declared.

## AUTHOR CONTRIBUTIONS

LZ, CW, and GL designed the study. LZ, ZZ, JL, WW, LF, MT, and JW performed the experiments, and LZ and HC performed analyses. LZ and GL wrote the manuscript with comments from all co‐authors.

## Supporting information

Supplementary Material

## Data Availability

The sequencing dataset for gut microbes has been submitted to the National Center for Biotechnology Information.
