# Supplementary material for: Gut microbiomes of bigheaded carps and hybrids provide insights into invasion: A hologenome perspective
Source: Evol Appl. 2020 Dec 22;14(3):735–45. doi: 10.1111/eva.13152 (PMC7980309; doi:10.1111/eva.13152)
Supplement: Supplementary file 1 — Supplementary Material [file EVA-14-735-s001.docx]

**Supporting Information**

**Table S1 The sample size in this study**

| Fish | Foregut | Hindgut |
| --- | --- | --- |
| Silver Carp | 19 | 30 |
| Bighead Carp | 11 | 26 |
| Hybrid BS | 16 | 23 |
| Hybrid SB | 19 | 26 |

The group names of species and hybrids and the names of gut sections are abbreviated as follows: B, S, BS, and SB represent Bighead Carp, Silver Carp, hybrids of Bighead (female) and Silver Carps (male), hybrids of Silver (female) and Bighead Carps (male), respectively whereas F and H represent foreguts and hindguts, respectively.

**Table S2 The mean abundance of the predominant genera in these Carp groups.**

Uncl, referred to the unclassified genus. B, S, BS, and SB, represent Bighead Carp, Silver Carp, hybrids of Bighead (female) and Silver Carps (male), and hybrids of Silver (female) and Bighead Carps (male) , respectively whereas F and H represent foreguts and hindguts, respectively.

**Table S3 Multiple comparison (Dunn-Sidàk correction) on the abundance of Cyanobacteria and Bacteroidetes in the foregut among groups (species)**

The one-way ANOVA showed significant difference among the pairwise unweighted Unifrac distance between the foregut and hindgut within each group (species). Then, we select the Dunn-Sidàk correction to make the multiple comparisons. SEM, Standard Error of Mean. B, S, BS, and SB, represent Bighead Carp, Silver Carp, hybrids of Bighead (female) and Silver Carps (male), and hybrids of Silver (female) and Bighead Carps (male), respectively.

**Figure S1 Gut bacterial compositions (phylum level) in pond-reared Bigheaded Carps.** B, S, BS, and SB, represent Bighead Carp, Silver Carp, hybrids of Bighead (female) and Silver Carps (male), and hybrids of Silver (female) and Bighead Carps (male), respectively. F and H represent foreguts and hindguts, respectively.

**Figure S2 Proportions of sequences at major bacterial phyla and significant differences in foreguts or hindguts and in different species group.** B, S, BS, and SB, represent Bighead Carp, Silver Carp, hybrids of Bighead (female) and Silver Carps (male), and hybrids of Silver (female) and Bighead Carps (male), respectively. The one-way ANOVA was used to test the significant difference in the main phyla within the same gut section among groups (species).

**Figure S3 Growth performances of Bigheaded Carps and hybrids in aquaculture ponds.** **A**. body weight (gram). **B,** body length (cm). **C**, body height (cm). **D**, body width (cm). One-Way ANOVA test was used to test the significant difference among species groups. LSD was used to perform multiple comparison. B, S, BS, and SB, represent Bighead Carp, Silver Carp, hybrids of Bighead (female) and Silver Carps (male), and hybrids of Silver (female) and Bighead Carps (male), respectively.
